# Transcriptome Sequencing Reveals That Curcumin Protects Leghorn Chicken Cardiomyocytes from Heat Stress-Induced Iron Dysregulation

**DOI:** 10.3390/ani16142200

**Published:** 2026-07-15

**Authors:** Meng Bian, Xiaojuan Zeng, Dingding Zhang, Jiapei Bu, Dingping Bai

**Affiliations:** Fujian Key Laboratory of Traditional Chinese Veterinary Medicine and Animal Health, College of Animal Sciences, Fujian Agricultural and Forestry University, Fuzhou 350002, China; bm321478965@163.com (M.B.); 15880556831@163.com (X.Z.); zhangdingpro@163.com (D.Z.); fqzimnw44@163.com (J.B.)

**Keywords:** cardiomyocytes, curcumin, Fe-S cluster, heat stress, heme, homeostasis, iron

## Abstract

The purpose of this study was to offer a theoretical foundation for the use of curcumin in poultry systems for anti-stress responses. Fourteen-day-old SPF Leghorn embryonic eggs were isolated and digested to produce primary cultured cardiomyocytes for this investigation. The primary cultured cardiomyocytes were divided into three groups: the CK group, serving as the control check; the HS group, which underwent a heat stress challenge; and the HS_Cur group, which was pre-treated with curcumin prior to experiencing heat stress. Twenty hours prior to the heat stress phase, the HS_Cur group received 15 μmol/L of curcumin. The culture medium’s cells and supernatant were extracted. Curcumin has been shown to protect cells against heat stress by drastically inhibiting the release of CK-MB and LDH, reducing the generation of MDA, and improving the potential of the mitochondrial membrane. Heat stress may alter cell proliferation, the base repair process, and cause proteotoxic stress, according to pathway enrichment analysis. Several iron metabolism-related pathways were both shown in CK vs. HS and HS vs. HS_Cur comparisons. Curcumin can promote heme synthesis and improve iron storage and antioxidant capacity by suppressing the expression of FTL, MAP1LC3C, and FXN and increasing the expression of SLC7A11, FTH1, FECH, SQSTM1, and NQO1, according to our subsequent analysis of iron metabolism-related DEGs. Curcumin may protect cells from the toxicity of LIP by inhibiting the expression of ferritinophagy-related genes and increasing the expression of heme production, iron storage, and antioxidant-related genes.

## Simple Summary

We investigated the protective impact of curcumin on cardiomyocytes during heat stress using RNA-seq. Our research may offer fresh insight into the harm that heat stress causes, which could be the foundation for the application of curcumin in poultry. Pathway enrichment research suggests that heat stress can affect cell proliferation, the base repair process, and induce proteotoxic stress. By suppressing the expression of genes linked to ferritinophagy and boosting the expression of genes associated with heme synthesis, iron storage, and antioxidants, curcumin may shield cells against the toxicity of labile iron pool (LIP). Curcumin could increase heme production and enhance iron storage capacity to shield cardiomyocytes from heat stress. The results of this study have significant ramifications for both animal welfare and animal productivity.

## 1. Introduction

One of the most important environmental limitations in poultry systems is heat stress (HS), which directly reduces growth performance, feed efficiency, and survivorship and eventually results in significant financial losses [1,2]. By controlling their cardiovascular systems to lower their body temperature, poultry adjust to hot environments. Heat stress causes a persistent overproduction of reactive oxygen species (ROS) in the heart, which is the primary organ responsible for blood circulation and body temperature regulation. The cardiomyocytes’ nucleic acids, proteins, and lipids are harmed by this high ROS, which induces myocardial remodeling and heart failure [3]. Research demonstrates that in fast-growing chickens, heat stress dramatically lowers heart weight by enhancing apoptosis and reducing cell cycle activity [4]. A relatively small heart may explain why modern fast-growing chickens are susceptible to heat stress [4]. In the meantime, heat stress causes a breakdown in cellular homeostasis and thermoregulatory control, which results in severe cardiovascular loads [5]. This evidence may suggest a connection between heat tolerance and heart health. As the primary pathogenic element in the development of fatal heart failure, cardiomyocytes are essential. Additionally, they participate in processes like ferroptosis and autophagy [6].

Heme is an iron containing molecule, and its homeostasis is essential for the health of cardiomyocytes. The study discovered that the heart failure model’s hemoglobin levels had significantly increased [7]. The degradation of heme was thought to be an important source of the LIP, which causes ferroptosis [8]. Mitochondria are the organelles responsible for heme synthesis. δ-aminolevulinate synthase (ALAS) and ferrochelatase (FECH) are the rate-limiting enzymes in heme biosynthesis [9,10]. Porphyrins are transported to the mitochondria by ATP-binding cassette transporter B6 (ABCB6), where they are utilized for heme synthesis and energy production [11]. Inhibition of ALAS2 and FECH expression will cause lack of heme and mitochondrial iron overload [9,12]. The preservation of mitochondrial iron homeostasis depends on the ATP-binding cassette transporter ABCB8 [13]. Research has demonstrated that malfunction of ABCB8 can cause iron excess in cardiomyocytes, which in turn can cause ferroptosis [14]. ABCB10 and mitochondrial ferritin-1 (SLC25A37) take part in the uptake and utilization of iron in mitochondria, and in cardiomyocytes, they work with iron chelatase and mitochondrial ferritin-1 (SLC25A37) to synthesize heme [15,16]. Iron is delivered to the mitochondria by NCOA4-mediated ferritinophagy, facilitating the production of heme and iron-sulfur (Fe-S) clusters [17].

Oxidative stress always coexists with heat stress [18]. Curcumin is a phenolic compound of turmeric, which has been proven to be an excellent anti-oxidant [19]. Curcumin has been shown in studies to alleviate unfavorable remodeling in the complicated cardiovascular environment, reduce myocardial cell damage, restrict fibrosis, and block inflammatory signaling [20]. However, nothing is known about whether curcumin can lessen the harm that heat stress causes to Leghorn cardiomyocytes by controlling iron metabolism.

Here, we used RNA-seq to investigate curcumin’s protective effect on cardiomyocytes during heat stress. Curcumin could increase heme production and enhance iron storage capacity to shield cardiomyocytes from heat stress. Our study may provide a new insight of the damage caused by heat stress. Additionally, we verified that curcumin protects cardiomyocytes from heat stress, which could serve as a basis for curcumin’s use in poultry.

## 2. Materials and Methods

### 2.1. Isolation and Heat Stress Model of Primary Chicken Cardiomyocytes

All animal experiments received approval from the Institutional Animal Care and Use Committee of Fujian Agriculture and Forestry University, China. Every procedure was conducted strictly following the committee’s established regulations and guidelines. Fourteen-day-old specific pathogen-free (SPF) Leghorn embryonated eggs were obtained from Sparfas Poultry Company (Jinan, China). The eggs were opened in a bio-clean environment. Ventricular tissue was then cut into pieces and washed four times with pre-cooled PBS. The tissue underwent digestion five times with pre-warmed (37 °C) 0.05% trypsin-EDTA (Gibco, Grand Island, NY, USA) for 5 min each; the first digestion was discarded. The remaining digests were centrifuged at 1000 rpm for 10 min. The precipitate was re-suspended in DMEM/high glucose (Hyclone, Logan, UT, USA) supplemented with 20% FBS (Gemini, Montgomery, AL, USA) and 0.1 mmol/L 5-Bromo-2-deoxyuridine (BrdU, Sigma, St. Louis, MO, USA). Cells were initially cultured in plates in a humidified incubator (Wuxi Marite Technology Co., Ltd., Wuxi, China) with 5% CO_2_ at 37 °C for 2 h. Afterwards, they were transferred to new culture plates and cultivated for 48 h in DMEM/high glucose containing 20% FBS and 0.1 mmol/L BrdU.

The primary cultured cells were divided into three groups: the CK group (control check), the HS group (heat stress challenge), and the HS_Cur group (pre-treated with curcumin before heat stress). Previous studies have demonstrated that treatment with 15 μmol/L curcumin for 20 h effectively induces its biological effects without inducing excessive cytotoxicity [21]. Therefore, this condition was selected for subsequent experiments. The HS_Cur group received 15 μmol/L curcumin (Sigma, St. Louis, MO, USA) 20 h before the heat stress phase. At the start of heat stress, both HS and HS_Cur groups were placed in a humidified incubator with 5% CO_2_ at 43 °C for 4 h, while the CK group remained at 37 °C with 5% CO_2_ for 4 h. Afterward, cells and the culture media supernatant were collected for further analysis.

### 2.2. Immunofluorescence

Isolated cells were seeded onto cell slides pre-installed in 6-well plates and subjected to the same protocol as the control group. After cell fixation, the samples were sent to Wuhan Savill Biotechnology Co., Ltd. Wuhan, China. for testing.

### 2.3. Detection of Cardiomyocytes-Related Enzymes

Cells and the culture media supernatant were collected to measure the activity of enzymes related to cardiomyocyte injury. Lactate dehydrogenase (LDH) and creatine kinase-myocardial band (CK-MB) activities were determined following the instructions provided with commercial kits (Nanjing Jiancheng Bioengineering Co., Ltd., Nanjing, China). Each set of data comes from three independent biological replicate experiments. Each sample was tested three times consecutively. The release ratio was calculated by dividing the enzyme activity in the culture media supernatant by the activity within the cells.

### 2.4. Analysis of Mitochondrial Membrane Potential (Δψm)

Apoptosis detection was performed using JC-1 Apoptosis detection kit (Keygen biotechnology Co., Ltd., Nanjing, China), with cell staining strictly conducted according to the manufacturer’s instructions. Select three independently cultured biological replicates. For each replicate, cells were cultured separately and harvested independently after treatment. The cell samples were analyzed using a flow cytometer (BD Accuri^TM^, Ann Arbor, MI, USA). Two independent computer-based data acquisitions were conducted separately, with 10,000 cell events measured per sample.

### 2.5. Assessment of Gsh and Gssg Content

GSH and GSSG were determined using GSH and GSSG detection kit, with all procedures strictly following the manufacturer’s instructions (Beyotime Biotechnology Co., Ltd., Shanghai, China). Three independent cell culture batches were selected. For each biological replicate sample, the lysate was divided into three portions and the protein concentrations in each group were measured using the BCA protein detection kit as instructed (Beyotime Biotechnology Co., Ltd., Shanghai, China).

### 2.6. Evaluation of Mda Content

The determination of MDA content was performed using the MDA assay kit, strictly following the procedures specified in the manufacturer’s instructions (Nanjing Jiancheng Bioengineering Co., Ltd., Nanjing, China). All MDA content data were derived from three independent cell culture batches. Each batch sample was tested with three technical replicate wells. The BCA protein detection kit was used to measure the protein concentration in each group according to the manufacturer’s instructions (Beyotime Biotechnology Co., Ltd., Shanghai, China).

### 2.7. RNA Extraction, Library Preparation, and Transcriptome Sequencing

The extraction of total RNA was performed using TRIzol reagent (Invitrogen, Carlsbad, CA, USA) in strict accordance with the manufacturer’s instructions. To obtain mRNA molecules with Poly-A tails, oligo-dT-attached magnetic beads were used to purify the extracted total RNA, followed by enzymatic digestion of the purified mRNA with fragmentation buffer. The first strand of cDNA was synthesized using random hexamer primer and reverse transcriptase, followed by the synthesis of the second strand of cDNA using DNA polymerase and RNase H. The synthesized cDNA products were purified using the QIAquick PCR Purification Kit (Qiagen, Hilden, Germany). The purified cDNA fragments underwent sequential steps including end repair, addition of a single’ A’ base, and adapter ligation, followed by further purification and final cDNA library construction via PCR enrichment. Sequencing of the library was performed by Guangzhou Gene Denovo Bioinformatics Technology Co., Ltd., Guangzhou, China. on an Illumina^®^ HiSeq 2500 platform.

### 2.8. Quality Control, Mapping, and Functional Annotation of Sequence Data

The quality assessment of the raw reads was performed using the FastQC [22]. To obtain high-quality, clean reads suitable for subsequent analyses, raw reads containing adapter sequences, those with an unknown base percentage exceeding 10%, or those failing to meet base quality standards were filtered out. Next, the qualified reads were aligned to the ribosomal database using the Bowtie tool to remove rRNA sequences. The remaining reads were then aligned to the reference genome (*Gallus gallus*) using the TopHat2 [23]. Following the alignment, the Cufflinks reference annotation-based transcript (RABT) assembly algorithm was employed to splice and assemble the successfully aligned reads into candidate transcripts, ultimately yielding the complete transcriptome assembly [24]. For functional enrichment analysis using Gene Ontology (GO) and pathway enrichment analysis using the Kyoto Encyclopedia of Genes and Genomes (KEGG), the statistical significance of both analyses was determined based on *p*-values.

### 2.9. RNA-Seq Data Analysis

In this study, DEseq2 was employed to analyze the read numbers mapped to each gene. Based on the gene length and the total number of reads aligned to the gene region, the FPKM (fragments per kilobase of transcript per million mapped reads) value for each gene was calculated. Differential expression analysis was performed using DEseq2, and differentially expressed genes (DEGs) were identified by screening with the following criteria: a corrected *p*-value of <0.05 and |fold change| > 1.2.

### 2.10. Real-Time Pcr Assay

cDNA reverse transcription was performed using the GoScript™ Reverse Transcription System (Promega, Madison, WI, USA), with all procedures conducted in accordance with the manufacturer’s instructions. Quantitative gene expression levels were achieved through real-time quantitative PCR using the CFX96 Touch Deep Well Real-Time PCR Detection System (Bio-Rad, Hercules, CA, USA). Amplification of target genes was performed with the specific primers listed in Table 1, with β-actin selected as the internal reference gene. The reaction mixture contained 6.25 μL of 2× GoTaq^®^ qPCR Master Mix (Promega, Madison, WI, USA), and operations were strictly carried out according to the manufacturer’s protocol. Each sample was run in three parallel reaction wells. The qRT-PCR protocol consisted of 3 min of pre-denaturation at 95 °C followed by 39 cycles of 95 °C for 20 s and 60 °C for 30 s. Fluorescence signals generated during the reaction were collected and analyzed using Bio-Rad CFX Manager 3.1, relative expression levels were calculated using the 2^−ΔΔCt^ method, with target gene expression data standardized relative to β-actin expression levels. To ensure data reliability, all primers were pre-validated using standard curves to confirm amplification efficiency (90–110%) and linear range (R^2^ ≥ 0.99), with melting curve analysis demonstrating a single peak, thereby verifying primer specificity meeting experimental requirements. Melting curves were plotted after completion of qPCR amplification. Primer information and candidate gene details are provided in Table 1.

### 2.11. Statistical Methods

Differences between groups were statistically analyzed using ANOVA. All experimental data were expressed as the mean ± SE of results from three independent experiments. To enhance the precision of testing, the Tukey method was employed. A *p*-value less than 0.05 was considered statistically significant for inter-group differences. All statistical analyses in this study were performed using SPSS 20.0.

## 3. Results

### 3.1. Isolation, Extraction and Identification of Chicken Embryo Cardiomyocytes

**Figure 1:**
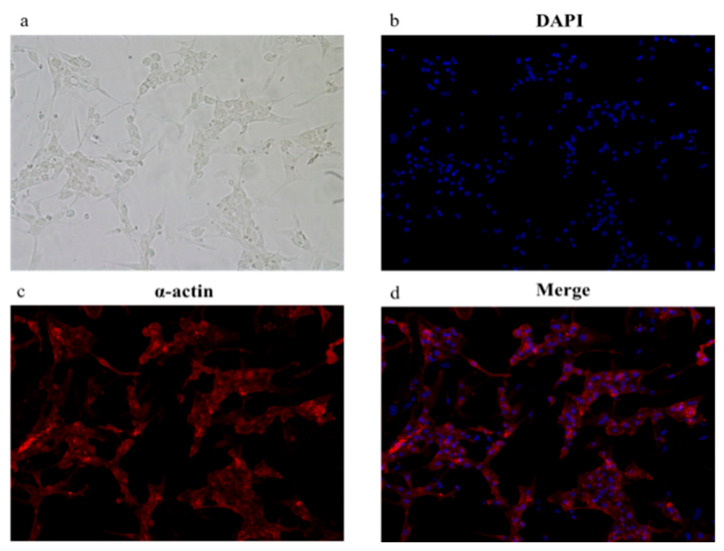
Immunofluorescence results. (**a**) Morphology of cardiomyocytes under light microscopy (400×); (**b**) DAPI-stained nuclei (400×); (**c**) α-actin immunofluorescence staining (400×); (**d**) DAPI and α-actin fluorescence overlap.

The immunofluorescence results for α-actin are shown in Figure 1c; α-actin is specific to muscle cells. The isolated chicken embryonic cardiomyocytes are spindle-shaped, and when the cells are aggregated, a pulsating cell population can be observed (Figure 1a). The blue fluorescence in the figure represents DAPI-labeled nuclei, while the red fluorescence indicates α-actin protein (Figure 1b,c). As shown in the figure, most cells exhibit strong red fluorescence, suggesting that the isolated cells are predominantly cardiomyocytes.

### 3.2. The Protective Impact of Curcumin on Cardiomyocytes Under Heat Stress

**Figure 2:**
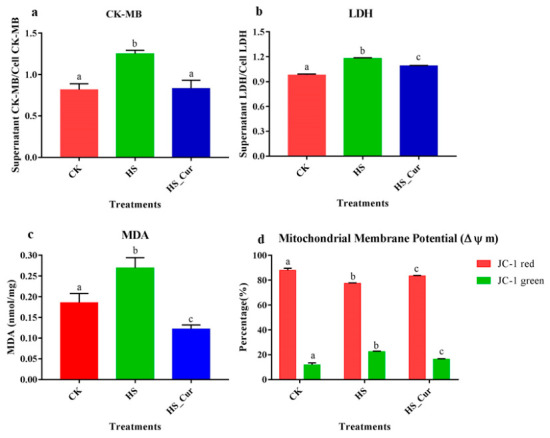
The protective effect of curcumin on cardiomyocytes under heat stress. (**a**) Release ratio of CK-MB; (**b**) release ratio of LDH; (**c**) MDA; (**d**) mitochondrial membrane potential.

Here, we evaluated the preservative effect of curcumin on cardiomyocytes under heat stress by measuring the release ratio of CK-MB and LDH, the cellular MDA, and mitochondrial membrane potential (ΔΨm). As shown in Figure 2a,b, the release ratio of CK-MB and LDH was significantly enhanced in HS group compared to CK group. And compared with HS group, curcumin could significantly decrease the release ratio of CK-MB and LDH. MDA is a lipid peroxidation product, its quantity can assess the degree of lipid peroxidation. Compared to CK group, heat stress significantly increased the MDA level. And compared with the CK and HS group, curcumin significantly decreased the MDA level (Figure 2c). ΔΨm reflects the function of mitochondria, and the decrease in ΔΨm is a sign of early stage apoptosis. Compared to the CK group, ΔΨm was significantly decreased by heat stress. The decreased ΔΨm will be increased by curcumin (Figure 2d). Above all, curcumin can protect cardiomyocytes from heat stress.

### 3.3. Overview of RNA-Seq Results

**Figure 3:**
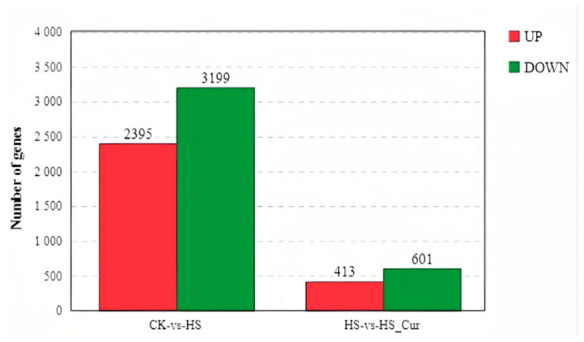
Differentially expressed genes statistics. The number of up-regulated and down-regulated genes in the CK vs. HS and HS vs. HS_Cur comparisons are summarized.

To investigate the protective mechanism of curcumin on cardiomyocytes under heat stress, we conducted a transcriptome analysis of cardiomyocytes in CK group, HS group and HS_Cur group. The results of the RNA-seq sequencing analysis are presented in Table 1 and Table 2, and Figure 2. The number of clean reads obtained from each sequencing library ranged from 44,118,656 to 60,782,244, with a Q20 value exceeding 97.53% (Table 2). After filtering out low-quality reads, adapter-contaminated sequences, and rRNA reads from the raw data, the clean reads exhibited a matching rate of up to 89.70% to the Gallus gallus genome (Table 3). Compared to the CK group, the HS treatment group showed a significant up-regulation trend in 2395 DEGs and a significant down-regulation trend in 3199 DEGs. In contrast, the HS_Cur group, demonstrated a significant up-regulation of 413 DEGs and a significant down-regulation of 601 DEGs compared to the HS treatment group (Figure 3).

### 3.4. Degs Functional Annotation

**Figure 4:**
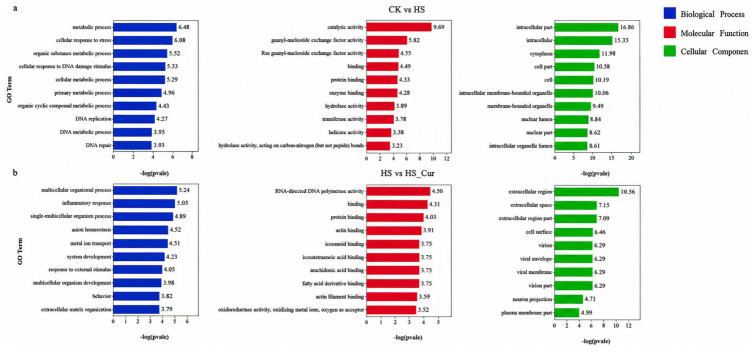
GO classification of DEGs. The blue column represents biological process; the red column represents molecular function; the green column represents cellular component. (**a**) CK vs. HS comparison; (**b**) HS vs. HS_Cur comparison.

The DEGs from CK vs. HS and HS vs. HS_Cur comparisons were annotated using the GO database (Figure 4). In the CK vs. HS comparison, metabolic process, cellular response to stress, organic matter metabolic process and cellular response to DNA damage stimulus were the most frequent terms in biological process. This may indicate that cellular metabolism was altered and DNA was damaged by heat stress. Catalytic activity, guanyl-nucleotide exchange factor function and activity of ras guanyl-nucleotide exchange factor were the top terms in molecular function. Intracellular part, intracellular and cytoplasm were the top terms in cellular component.

In the HS vs. HS_Cur comparison, multicellular organismal process, inflammatory response and the processes related to single-multicellular organism were the most prevalent terms in biological process; RNA-directed DNA polymerase function, binding activity, and protein binding were the top terms in molecular function; extracellular region, extracellular space and extracellular region part were the top terms in cellular component.

**Figure 5:**
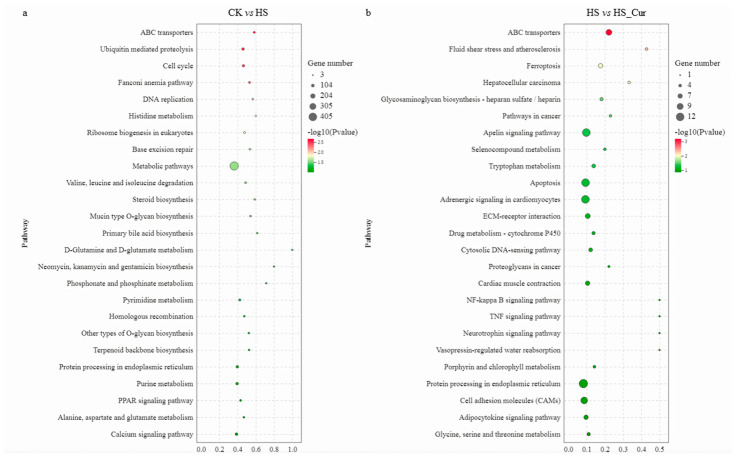
The top 25 pathways in KEGG enrichment by *p*-value. (**a**) CK vs. HS comparison; (**b**) HS vs. HS_Cur comparison.

Pathway enrichment analysis was conducted based on the KEGG pathway database (Figure 5). In CK vs. HS comparison, Ubiquitin-mediated proteolysis, eukaryotes ribosome biogenesis, and endoplasmic reticulum protein processing were related to proteotoxic stress caused by heat stress [25,26,27], which were shown in the top 25 KEGG list. Cell cycle, DNA replication and base excision repair were also shown in the list, which indicated that cell proliferation and base repair will be altered by heat stress. Terpenoid backbone biosynthesis was also shown in the KEGG list, which is related to the synthesis of CoQ and GPX4 and could increase the resistant ability of cell to iron.

In HS vs. HS_Cur, ferroptosis, porphyrin and chlorophyII metabolism were involved in iron metabolism. The KEGG analysis also identified pathways related to fluid shear stress and atherosclerosis, adrenergic signaling in cardiomyocytes, and cardiac muscle contraction, which were related to the function of cardiomyocytes.

### 3.5. Identification of the DEGs Related to Iron Homeostasis

Iron metabolism-related pathways were shown in the KEGG list in both comparisons. Ferroptosis, an iron-catalyzed form of regulated necrosis, results from the excessive peroxidation of polyunsaturated fatty acids (PUFAs) [28]. The ferroptosis pathway enrichment was statistically significant between the HS and Cur groups (*p* < 0.05).

**Figure 6:**
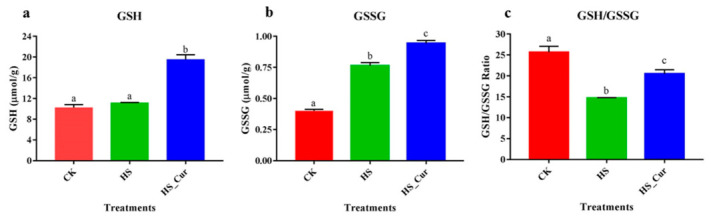
Evaluation of GSH and GSSG content (**a**) GSH; (**b**) GSSG; (**c**) GSH/GSSG.

GSH is the co-factor of GPX4, which is a central enzymatic component of the antioxidant system [29]. Compared with the CK group, SLC7A11 was down-regulated and GCLC was up-regulated by heat stress, which resulted in no significant difference in the content of GSH in the HS group (Table 4 and Figure 6). Compared with the HS group, SLC7A11 was up-regulated by curcumin, which resulted in a significant increase in GSH (Table 4 and Figure 6). Mevalonate pathway produces isopentenyl pyrophosphate (IPP) and farnesyl diphosphate (FPP) [30]. IPP could provide selenium for the active center of the GPX4 [31]. FPP is a vital molecule for respiratory chain and resisting to ferroptosis [31]. Compared with the CK group, the expression of several genes involved in the mevalonate pathway (HMGCR, HMGCS1, MVK, MVD and PMVK) were significantly decreased in the HS group. There was no notable difference in the expression of mevalonate pathway-related genes between HS group and HS_Cur group.

Next, we paid attention to several iron metabolism-related genes. FTL and FTH1 are the compositions of ferritin, which is utilized to store excess iron [32]. When cellular iron is depleted, FTL knockout cells inhibit ferritin degradation by decreasing interactions with NCOA4 [33]. MAP1LC3B2 and MAP1LC3C participate in ferritinophagy [33]. Currently, FPN stands as the only known protein capable of exporting iron [34]. Compared to the CK group, heat stress could increase the iron releasing by up-regulating the expression of NCOA4. Meanwhile, heat stress could decrease the iron content by inhibiting the expression of MAP1LC3B2 and promoting the transcription of FPN. We found that curcumin could up-regulate FTH1 and down-regulate FTL and MAP1LC3C to increase iron storage ability and inhibit ferritin degradation.

ISCA2 and IBA57 is the composition of ISA complex, which participates in the Fe/S assembly in mitochondria [35]. NFU1 transfers [4Fe-4S] cluster from the ISA complex to client protein to protect [4Fe-4S] cluster from oxidative damage [36]. Frataxin (FXN) functions as both an iron oxidase and an iron chaperone, regulating iron homeostasis and providing an iron source for heme biosynthesis [37]. In the curcumin-treated group, FXN was significantly down-regulated compared to HS group. The inhibition of heme and [4Fe-4S] cluster by heat stress may increase the LIP and disturb the distribution of intracellular iron.

LIP is one of the sources for ROS, which will be eliminated by Nrf2 target proteins. Keap1 is the negative regulator of Nrf2 [38], which was up-regulated by heat stress. Compared to HS group, Keap1 was up-regulated by curcumin. SQSTM1 could activate Nrf2 by inactivation of Keap1 [39]. SQSTM1 was down-regulated by heat stress compared to CK group. While SQSTM1 was up-regulated by curcumin compared to HS group. NQO1 is the negative regulator of ferroptosis [40]. It was up-regulated by curcumin compared to the HS group.

### 3.6. Verification of Degs by Qpcr

**Figure 7:**
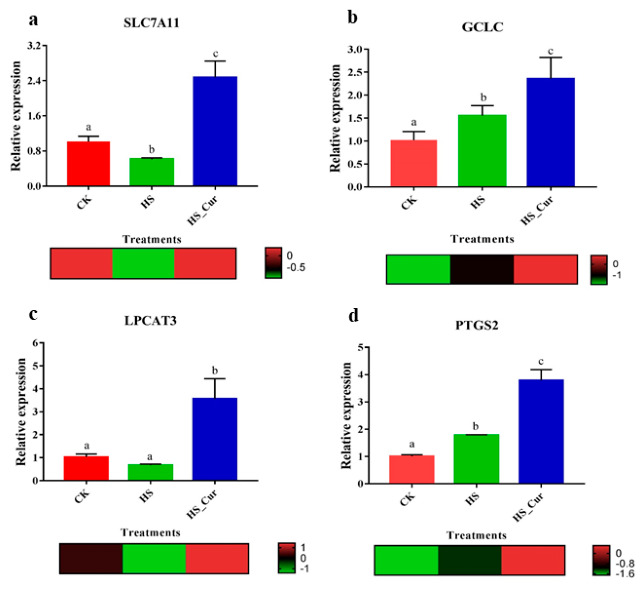
Expression levels of candidate genes by RT-qPCR and RNA-seq. The heat map shows the FPKM values for the four selected candidate genes. GAPDH was used as the reference gene. (**a**) SLC7A11; (**b**) GCLC; (**c**) LPCAT3; (**d**) PTGS2.

To confirm the accuracy, reliability, and experimental reproducibility of the results obtained from this transcriptome sequencing analysis, we selected four genes to conduct RT-qPCR validation (Figure 7). Using RT-qPCR technology, we analyzed the expression profiles of these four candidate genes and found that the results were consistent with those obtained from RNA-seq sequencing, demonstrating our transcriptome analysis.

## 4. Discussion

Apoptosis, inflammation, autophagy, heat shock response, and oxidative stress are the main topics of many studies on heat stress [18,41,42,43,44]. Iron homeostasis receives less attention. In this paper, we investigated the protective impact of curcumin on heat-stressed cardiomyocytes using transcriptome analysis. ABC transporters participate in mitochondrial iron transport, which was the most significantly enriched term in both comparisons [45,46,47]. In addition to taking part in tissue pathological processes, the ABC transporter shields the heart from oxidative stress damage brought on by medicines and harmful chemicals [48]. Ferroptosis-related terpenoid backbone biosynthesis was displayed in the KEGG list in the CK vs. HS comparison [49,50]. Iron metabolism-related ferroptosis, porphyrin, and chlorophyll II metabolism were also displayed in both comparisons. Ferroptosis is a unique process of cell death involved in high iron and lipid peroxidation [51]. Porphyrin and chlorophyII metabolism is a pathway that is involved in the synthesis of heme [11]. The majority of iron in the human body is used for the creation of Heme- and Fe-S cluster cofactors. Iron metabolism may potentially be impacted by heme production. We assumed that heat stress may disturb iron homeostasis, and curcumin can protect cells from toxicity caused by free iron [8].

We concentrated on the DEGs associated with iron metabolism in our DEG analysis. Research has demonstrated that mitochondrial iron excess is associated with altered Fe-S cluster and heme biogenesis [52]. Thus, we also focused on DEGs associated with heme production and Fe-S clusters. Under heat stress, we discovered that five DEGs relevant to heme synthesis were down-regulated, including the rate-limiting enzymes FECH and ALAS2 [53]. Heat stress inhibited NFU1, which is necessary for transporting Fe-S to client protein [54]. Heat stress may encourage additional iron to be imported into mitochondria by interfering with the production of heme and Fe-S to client protein. In our investigation, we discovered that heat stress increased the expression of ferritinophagy-related NCOA4, SLC25A37, and mitochondrial iron importer ABCB10. Iron overload in the cytosol and mitochondria may result from the overexpression of ABCB10, SLC25A37, and NCOA4. These will cause ferritinophagy-related MAP1LC3B2 to be down-regulated and iron exporter ABCB8 and ferroportin to be up-regulated. Curcumin could up-regulate the expression of FECH and down-regulate FXN to promote the synthesis of heme. Curcumin may promote FTH1 expression in the cytoplasm to boost iron storage capacity. Moreover, curcumin may prevent ferritinophagy by down-regulating the expression of FTL and MAP1C3C. Heat stress has been shown to enhance iron buildup and change the expression of iron regulatory proteins [55]. This result is similar with ours, while the cellular concentration of iron in our study needs further investigation.

The primary source of hydroxyl radicals, which might result in lipid peroxidation, is an excess of iron [56]. Lipid-based reactive oxygen species are the hallmark of ferroptosis, a novel type of cell death [57]. For cells to survive under heat stress, they must be able to get rid of lipid-based reactive oxygen species. Thus, we also examined the DEGs associated with antioxidants. There was no discernible change in the GSH level as a result of heat stress repressing the expression of SLC7A11 and increasing the representation of GCLC. However, heat stress considerably raised the MDA level, indicating that lipid peroxidation was higher in the heat-stressed group than in the control group. Polyunsaturated fatty acids are shielded from lipid peroxidation by GPX4 [29]. Ferroptosis, LPO buildup, and lipid peroxidation are caused by GPX4 deficiency. Curcumin can trigger the Nrf2 protein to reach the nucleus and promote the production of antioxidant genes, as we have shown in earlier research [19]. According to recent research, Nrf2 regulates a number of genes associated with heme synthesis (such as ABCB6 and FECH), iron storage (such as ferritin heavy chain FTH1 and ferritin light chain FTL), and iron transport (such as iron transporter FPN) [58,59]. Curcumin could up-regulate SLC7A11 to increase the GSH level and inhibit the production of MDA. In the meantime, curcumin could increase the number of activated Nrf2 by increasing the expression of SQSTM1, which will induce the expression of NQO1, FECH, FTH1.

## 5. Conclusions

**Figure 8:**
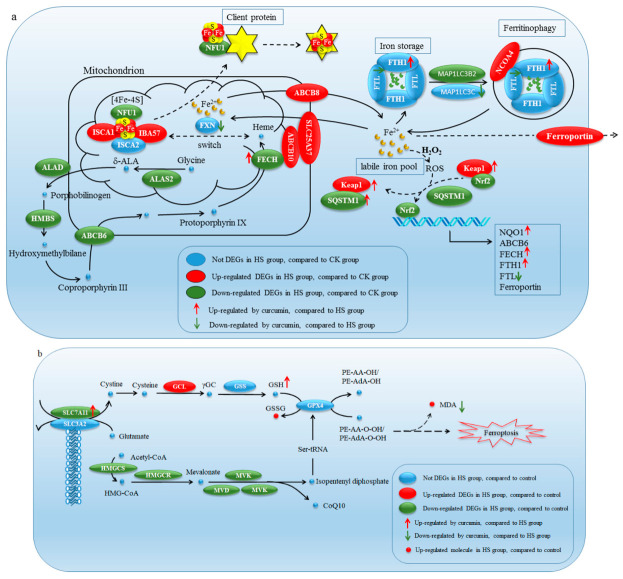
The explanation of the protective effect of curcumin on cardiomyocytes under heat stress from the perspective of mRNA level. (**a**) Heme and Fe-S with iron homeostasis; (**b**) GPX4 and lipid peroxidation. The blue oval represents that there was no significant difference between CK and HS group; the red oval represents that it was up-regulated in HS group compared to CK group; the green oval represents that it was down-regulated in HS group compared to CK group; the red arrow represents that it was up-regulated in HS_Cur group compared to HS group; the green arrow represents that it was down-regulated in HS_Cur group compared to HS group.

On the one hand, we discovered that heat stress reduced the representation of genes linked to heme production. These may decrease the utilization of iron from LIP. On the other hand, the suppression of heme and NFU1 expression may have led to a shortage of heme and prevented Fe-S from reaching client protein, which could trigger iron starvation stress. IRP1 and ferritinophagy-related NCOA4 were up-regulated in heat stress group, which elevates LIP. LIP is one of the sources of ROS and could induce ferroptosis. The Nrf2 activity may be suppressed by activating the representation of KEAP1 and inhibiting the representation of SQSTM1 under heat stress (Figure 8a). We discovered that heat stress may reduce GPX4 function by inhibiting the production of genes linked to the mevalonate pathway, which may result in lipid peroxidation. (Figure 8b).

Curcumin could enhance the heme level and iron utilization by suppressing the expression of FXN and increasing the expression of FECH. Curcumin altered ferritin’s composition, which may improve iron storage capacity and prevent ferritinophagy. Curcumin may prevent ferroptosis by increasing GSH production and SQSTM1 and NQO1 expression (Figure 8). Direct measurements of parameters like LIP and GPX4 activity are still necessary to conclusively establish the functions of these processes, even if our data strongly suggest their existence. Future studies will directly measure key functional parameters to conclusively confirm this mechanism.

## Figures and Tables

**Table 1:** Primer sequences for target genes and internal reference genes required for qRT-PCR.

Gene	Primer Sequence (5′→3′)
β-actin	(F)GTGGATCAGCAAGCAGGAGT
	(R)ATCCTGAGTCAAGCGCCAAA
caspase3	(F)GATGCTGCAAGTGTCAGA
	(R)ATCGCCATGGCTTAGCA
caspase9	(F)TCAGACATCGTATCCACCA
	(R)AAGTCACAGCAGGGACA
HSF1	(F)GCTCATTCCATGCCGAAATA
	(R)GGATGAATCGGGGGAGTAG
HSF2	(F) ATGGCCAGAGTTTCTTGGTG
	(R) GGACAACTTTACGGAAGCCA
HSF3	(F) TCAAGCAATGTTATCTGGGAA
	(R) CTTCAAACAACGAAAGCAGAG
HSF4	(F) CATCGCTGTATGTTCCATCT
	(R) ACTGGTTGTTATCAGTCGAA
HSP70	(F)CCAAACAAACACAGACCTTC
	(R)CGTTCAGGATACCATTAGCA
HSP90aa	(F)AAAAGAGGGCTTAGAGCTTC
	(R)CATTGTGGAGTTGTCTCTCA
HSP90ab	(F)CTCTCACCCTGCTGGAC
	(R)GGTAATGACAACCACCTTCT
*Cytb*-DNA	(F) CCTCACACTCATAGCCACCG
	(R) CCCCTCAGGCTCACTCTACT
Gapdh-DNA	(F) GGGGAAAGTCATCCCTGAGC
	(R) TTGGCTGGTTTCTCCAGACG

**Table 2:** Summary of the RNA-seq data collected from CK, HS and HS_Cur groups.

Sample Name	Raw Reads	Clean Read Number	Adapter (%)	Low-Quality (%)	Poly-A (%)	N (%)	Q20 (%)	GC (%)
CK-1	58,123,746	57,977,880 (99.75%)	32,440 (0.06%)	113,426 (0.20%)	0 (0.00%)	0 (0.00%)	855,3457,054 (98.11%)	4,621,415,178 (53.01%)
CK-2	50,523,024	50,339,438 (99.64%)	65,802 (0.13%)	117,262 (0.23%)		522 (0.00%)	7,391,027,586 (97.53%)	3,900,775,977 (51.47%)
CK-3	53,047,124	52,861,240 (99.65%)	50,118 (0.09%)	135,766 (0.26%)		0 (0.00%)	7,786,833,986 (97.86%)	4,139,385,618 (52.02%)
HS-1	49,843,686	49,668,590 (99.65%)	46,910 (0.09%)	128,186 (0.26%)		0 (0.00%)	7,317,180,832 (97.87%)	3,874,100,693 (51.82%)
HS-2	59,920,998	59,727,944 (99.68%)	51,386 (0.09%)	141,668 (0.24%)		0 (0.00%)	8,798,278,794 (97.89%)	4,683,940,374 (52.11%)
HS-3	44,118,656	43,964,594 (99.65%)	43,902 (0.10%)	110,160 (0.25%)		0 (0.00%)	6,482,605,394 (97.96%)	3,450,258,130 (52.14%)
HS_Cur-1	47,472,910	47,324,684 (99.69%)	38,214 (0.08%)	110,012 (0.23%)		0 (0.00%)	6,977,589,562 (97.99%)	3,740,338,975 (52.53%)
HS_Cur-2	54,398,710	54,215,084 (99.66%)	51,138 (0.09%)	132,488 (0.24%)		0 (0.00%)	7,987,638,682 (97.89%)	4,268,991,696 (52.32%)
HS_Cur-3	60,782,244	60,574,466 (99.66%)	59,056 (0.10%)	148,722 (0.24%)		0 (0.00%)	8,931,523,593 (97.96%)	4,769,054,275 (52.31%)

**Table 3:** Summary of clean reads mapped to reference genome from CK, HS and HS_Cur groups.

Sample Name	Total Reads	Multiple Mapped	Unique Mapped	Known Genes	Novel Genes	Total Genes
CK-1	57,031,154	1,318,452 (2.31%)	51,729,650 (90.70%)	15,185 (91.65%)	923 (98.09%)	16,108 (92.00%)
CK-2	49,379,568	1,075,082 (2.18%)	45,161,442 (91.46%)	15,159 (91.50%)	926 (98.41%)	16,085 (91.87%)
CK-3	52,159,982	1,127,740 (2.16%)	47,247,607 (90.58%)	15,136 (91.36%)	921 (97.87%)	16,057 (91.71%)
HS-1	48,847,168	1,030,944 (2.11%)	44,431,101 (90.96%)	15,040 (90.78%)	926 (98.41%)	15,966 (91.19%)
HS-2	58,463,770	1,295,756 (2.22%)	52,443,260 (89.70%)	15,188 (91.67%)	928 (98.62%)	16,116 (92.04%)
HS-3	43,283,996	911,348 (2.11%)	39,175,523 (90.51%)	14,987 (90.46%)	928 (98.62%)	15,915 (90.90%)
HS_Cur-1	46,659,624	985,291 (2.11%)	42,529,811 (91.15%)	15,095 (91.11%)	930 (98.83%)	16,025 (91.52%)
HS_Cur-2	53,466,102	1,168,828 (2.19%)	48,499,911 (90.71%)	15,146 (91.42%)	926 (98.41%)	16,072 (91.79%)
HS_Cur-3	59,775,552	1,285,962 (2.15%)	54,311,280 (90.86%)	15,203 (91.76%)	931 (98.94%)	16,134 (92.15%)

**Table 4:** Identification of the DEGs related to iron homeostasis.

Function	Gene Name	CK vs. HS		HS vs. Cur		Products
		log2FC	UP or DOWN	log2FC	UP or DOWN	
GSH synthase	SLC7A11	−0.399	DOWN	0.397	UP	solute carrier family 7 member 11
	GCLC	0.358	UP	0.113	N.S	glutamate-cysteine ligase catalytic subunit
Ser-tRNA isoprenylation	HMGCR	−0.406	DOWN	0.065	N.S	3-hydroxy-3-methylglutaryl-CoA reductase
	HMGCS1	−0.341	DOWN	−0.062	N.S	3-hydroxy-3-methylglutaryl-CoA synthase 1
	MVK	−0.371	DOWN	−0.228	N.S	mevalonate kinase
	MVD	−0.48	DOWN	0.166	N.S	mevalonate diphosphate decarboxylase
	PMVK	−0.665	DOWN	−0.065	N.S	phosphomevalonate kinase
Iron regulator	IRP1	0.474	UP	0.021	N.S	aconitase 1
Fe^2+^ storage	FTL	0.113	N.S	−0.265	DOWN	ferritin light chain
	FTH1	0.178	N.S	0.316	UP	ferritin heavy chain 1
Fe^2+^ release	NCOA4	0.428	UP	−0.063	N.S	nuclear receptor coactivator 4
	MAP1LC3B2	−0.263	DOWN	0.133	N.S	microtubule associated protein 1 light chain 3 beta 2
	MAP1LC3C	−0.273	N.S	−0.437	DOWN	microtubule associated protein 1 light chain 3 gamma
	FPN	0.346	UP	0.017	N.S	Ferroportin
heme synthase	ALAS2	−0.316	DOWN	0.186	N.S	5-aminolevulinate synthase 2
	ALAD	−0.721	DOWN	0.052	N.S	aminolevulinate dehydratase
	HMBS	−0.405	DOWN	0.013	N.S	hydroxymethylbilane synthase
	ABCB6	−0.523	DOWN	0.206	N.S	ATP binding cassette subfamily B member 6
	FECH	−0.334	DOWN	0.353	UP	ferrochelatase
	ABCB8	0.414	UP	−0.022	N.S	ATP binding cassette subfamily B member 8
	ABCB10	0.468	UP	0.098	N.S	ATP binding cassette subfamily B member 10
	SLC25A37	0.561	UP	0.094	N.S	solute carrier family 25 (mitochondrial iron transporter), member 37
Fe-S cluster synthesis	ISCA1	0.437	UP	−0.057	N.S	iron-sulfur cluster assembly 1
	IBA57	1.217	UP	−0.064	N.S	IBA57 homolog
	NFU1	−0.314	DOWN	−0.117	N.S	NFU1 iron-sulfur cluster scaffold
	FXN	−0.006	N.S	−0.415	DWON	frataxin
anti-oxidate	Nrf2	−0.523	DOWN	0.002	N.S	nuclear factor, erythroid 2 like 2
	KEAP1	0.526	UP	0.383	UP	kelch-like ECH-associated protein 1
	SQSTM1	−0.347	DOWN	0.277	UP	sequestosome 1
	NQO1	−0.253	N.S	0.528	UP	NAD(P)H dehydrogenase [quinone] 1 isoform 1

## Data Availability

The datasets used and/or analyzed during the current study are available from the corresponding author on reasonable request.

## Abbreviations

DEGs	differentially expressed genes
LIP	labile iron pool
ALAS	δ-aminolevulinate synthase
FECH	Ferrochelatase
ABCB6	ATP-binding cassette transporter B6
ABCB8	ATP-binding cassette transporter B8
ABCB10	ATP-binding cassette transporter B10
ALA	δ-aminolevulinic acid
SLC25A37	solute carrier family 25 (mitochondrial iron transporter), member 37; mitoferrin-1
FTH1	ferritin heavy chain
FTL	ferritin light chain
FPN	ferroportin
ARFs	ADP-ribosylation factors
ISC	iron-sulfur cluster
PUFAs	polyunsaturated fatty acids
IPP	isopentenyl pyrophosphate
FPP	farnesyl diphosphate
SLC7A11	solute carrier family 7 member 11
GCLC	glutamate-cysteine ligase catalytic subunit
HMGCR	3-hydroxy-3-methylglutaryl-CoA reductase
HMGCS1	3-hydroxy-3-methylglutaryl-CoA synthase 1
MVK	mevalonate kinase
MVD	mevalonate diphosphate decarboxylase
PMVK	phosphomevalonate kinase
NCOA4	nuclear receptor coactivator 4
MAP1LC3B2	microtubule associated protein 1 light chain 3 beta 2
MAP1LC3C	microtubule associated protein 1 light chain 3 gamma
ALAD	aminolevulinate dehydratase
HMBS	hydroxymethylbilane synthase
ISCA1	iron-sulfur cluster assembly 1
IBA57	IBA57 homolog
NFU1	NFU1 iron-sulfur cluster scaffold
FXN	frataxin
Nrf2	nuclear factor, erythroid 2 like 2
KEAP1	kelch-like ECH-associated protein 1
SQSTM1	sequestosome 1
NQO1	NAD(P)H dehydrogenase [quinone] 1 isoform 1
